# 伴1q21+及t（4;14）双打击多发性骨髓瘤的临床特征、治疗及预后

**DOI:** 10.3760/cma.j.cn121090-20250520-00238

**Published:** 2026-02

**Authors:** 瑛 郭, 妞 乔, 怡 陶, 坤明 刘, 皖雁 欧阳, 元昉 刘, 焰 王, 卫平 章, 坚青 糜

**Affiliations:** 1 上海交通大学医学院附属瑞金医院血液科，医学基因组学国家重点实验室，上海血液学研究所，国家转化医学研究中心（上海），上海 200025 Shanghai Institute of Hematology, State Key Laboratory of Medical Genomics, National Research Center for Translational Medicine at Shanghai, Ruijin Hospital Affiliated to Shanghai Jiao Tong University School of Medicine, Shanghai 200025, China; 2 九江市第一人民医院血液内科，九江 332000 Department of Hematology, The First People's Hospital of Jiujiang, Jiujiang 332000, China; 3 宿州市第一人民医院血液内科，宿州 234000 Department of Hematology, The First People's Hospital of Suzhou, Suzhou 234000, China; 4 上海市血液病基因编辑与细胞治疗重点实验室，上海 200025 Shanghai Key Laboratory of Gene Editing and Cell Therapy in Hematology, Shanghai 200025, China

**Keywords:** 多发性骨髓瘤, 1q21获得/扩增, t（4;14）, 造血干细胞移植, 抗体，单克隆, 预后, Multiple myeloma, 1q21 gain/amplification, t（4;14）, Hematopoietic stem cell transplantation, Antibodies, monoclonal, Prognosis

## Abstract

**目的:**

分析同时携带1q21拷贝数增加（1q21+）与t（4;14）的双打击多发性骨髓瘤（DHMM）患者的临床特征、治疗方案及预后，探讨改善该高危亚组患者预后的有效策略。

**方法:**

回顾性分析2014年9月至2024年9月上海交通大学医学院附属瑞金医院收治的96例同时携带1q21+与t（4;14）的初治DHMM患者的基线临床特征、预后及独立预后因素，Logistic回归模型分析诱导治疗方案、自体造血干细胞移植（auto-HSCT）等与微小残留病（MRD）阴性的相关性。

**结果:**

96例DHMM患者中位年龄62（36～79）岁，50例（52％）为R2-ISS分期Ⅳ期，11例（11％）合并del（17p），35例（36％）接受auto-HSCT，39例（41％）采用蛋白酶体抑制剂（PI）+免疫调节剂（IMiD）+抗CD38单克隆抗体（CD38Ab）三联诱导治疗，25例（26％）达MRD阴性的完全缓解（CR）。1q21+与t（4;14）克隆大小呈正相关（*r*＝0.46，*P*<0.001），但1q21+的克隆负荷显著低于t（4;14）。中位随访36（6～126）个月，中位无进展生存（PFS）期为26（95％*CI*：22～50）个月，预估中位总生存（OS）期为4.3（95％*CI*：2.1～6.4）年，22例（23％）患者发生髓外复发。多因素分析表明，初诊伴软组织髓外病变是PFS和OS的独立危险因素（*P*值均<0.05），del（17p）使PFS期缩短，MRD阴性使PFS期显著延长，三联诱导治疗（PI+IMiD+CD38Ab）和auto-HSCT均能提高MRD阴性率（*P*值均<0.05）。

**结论:**

同时携带1q21+和t（4;14）的DHMM患者呈侵袭性临床特征，预后不良。三联诱导治疗（PI+IMiD+CD38Ab）和auto-HSCT与MRD阴性、PFS改善相关，但患者实现长期生存仍具挑战。

## Abstract (en)

**Objective:**

To analyze the clinical characteristics, therapeutic regimens, and prognosis of double-hit multiple myeloma（DHMM）patients with concurrent 1q21 copy number gain（1q21+）and t（4;14）, and to explore effective strategies to improve outcomes of this high-risk subgroup.

**Methods:**

A retrospective analysis was performed on 96 newly diagnosed DHMM patients with both 1q21+ and t（4;14）admitted to Ruijin Hospital Affiliated to Shanghai Jiao Tong University School of Medicine from September 2014 to September 2024. Baseline clinical characteristics, prognosis, and independent prognostic factors were evaluated. A logistic regression model was used to analyze the correlation between induction regimens, autologous hematopoietic stem cell transplantation（auto-HSCT）, and minimal residual disease（MRD）negativity.

**Results:**

The median age of the 96 DHMM patients was 62（range: 36–79）years. Among them, 50 cases（52％）were at R2-ISS stage Ⅳ, 11 cases（11％）had concurrent del（17p）, 35 cases（36％）underwent auto-HSCT, 39 cases（41％）received triple-agent induction therapy with proteasome inhibitor（PI）, immunomodulatory drug（IMiD）and anti-CD38 monoclonal antibody（CD38Ab）, 25 cases（26％）achieved MRD-negative complete response（CR）. The clone size of 1q21+ was positively correlated with that of t（4;14）（*r*＝0.46, *P*<0.001）, while the clonal burden of 1q21+ was significantly lower than that of t（4;14）. With a median follow-up of 36（range: 6–126）months, the median progression-free survival（PFS）was 26（95％*CI*: 22–50）months, and the estimated median overall survival（OS）was 4.3（95％*CI*: 2.1–6.4）years. Extramedullary relapse occurred in 22 cases（23％）. Multivariate analysis showed that newly diagnosed extramedullary involvement of soft tissue was an independent risk factor for both PFS and OS（all *P*<0.05）. Del（17p）shortened PFS, whereas MRD negativity significantly prolonged PFS. Both triple-agent induction therapy（PI+IMiD+CD38Ab）and auto-HSCT improved the MRD-negative rate（all *P*<0.05）.

**Conclusion:**

DHMM patients with concurrent 1q21+ and t（4;14）exhibit aggressive clinical features and poor prognosis. Triple-agent induction therapy（PI+IMiD+CD38Ab）and auto-HSCT are associated with MRD negativity and improved PFS, but achieving long-term survival remains challenging for these patients.

近二十年来，初诊多发性骨髓瘤（NDMM）的治疗领域已取得显著进展。新药［包括蛋白酶体抑制剂（PI）、免疫调节剂（IMiD）、单克隆抗体等］及大剂量化疗联合自体造血干细胞移植（auto-HSCT）的应用显著改善了患者的生存。然而，仍有部分NDMM患者预后较差，主要由细胞遗传学异常（CA）驱动[1]。高危CA包括1q21拷贝数增加（1q21+）、t（4;14）、t（14;16）和del（17p）[2]，其中，1q21+是最常见的高危CA，在20％～50％的NDMM患者中可见[3]。1q21+可分为两种亚型：1q21获得（3个拷贝）和1q21扩增（>3个拷贝）。尽管有研究显示，携带1q21扩增的NDMM患者的预后较1q21获得患者更差，但这一结论仍存在争议[4]–[5]。值得注意的是，尽管新型疗法不断发展，1q21+的不良预后意义依然持续存在[6]。t（4;14）是11％～15％的NDMM患者的主要克隆事件[7]，该易位使成纤维细胞生长因子受体3（FGFR3）和核受体结合SET结构域蛋白2（NSD2）基因受免疫球蛋白重链（IgH）超级增强子调控[8]，导致FGFR3过度激活及NSD2表观遗传异常，进而驱动肿瘤细胞增殖。GRIFFIN、MASTER等多项关于新药联合auto-HSCT的临床研究显示，DaraVRD方案（达雷妥尤单抗+硼替佐米+来那度胺+地塞米松）及DaraKRD方案（达雷妥尤单抗+卡非佐米+来那度胺+地塞米松）联合auto-HSCT治疗可显著改善携带单一高危CA的NDMM患者的预后，使其无进展生存（PFS）期与无高危CA患者接近[9]。然而，携带≥2个高危CA的双打击多发性骨髓瘤（DHMM）患者在全球临床试验及本中心真实世界研究中均呈现较差的预后结局[9]–[10]。本团队最近研究进一步表明，DHMM患者发生髓外复发（sEMD）的风险显著升高[11]。从细胞遗传学角度分析，DHMM具有明显的异质性，其中1q21+与t（4;14）同时存在是最常见的亚型，据文献报道，该亚型患者的中位总生存（OS）期仅4.4年（20例）[12]。

尽管DHMM在当前治疗背景下具有重要临床意义，但目前关于该亚组患者的大样本、真实世界数据仍较为有限。本研究回顾性分析2014年至2024年单中心96例同时携带1q21+和t（4;14）的DHMM患者，系统评估其基线临床特征、1q21+与t（4;14）克隆负荷、治疗方案选择、微小残留病（MRD）状态及预后，旨在深入阐明这类高危患者的疾病特征，为改善其临床预后提供循证依据。

## 病例与方法

1. 病例：回顾性分析上海交通大学医学院附属瑞金医院2014年9月至2024年9月收治的96例同时携带1q21+与t（4;14）的DHMM患者。髓外病变（EMD）定义为在符合多发性骨髓瘤（MM）诊断标准的基础上，合并浆细胞瘤骨髓外浸润，其中突破骨皮质侵犯周围软组织者定义为骨旁髓外病变（EM-B），由血源性播散导致远离骨骼的解剖部位出现软组织肿瘤者定义为软组织髓外病变（EM-E），若患者同时存在EM-B和EM-E，则归为EM-E组。本研究已通过上海交通大学医学院附属瑞金医院伦理委员会审批（审批号2025-235），并获得患者的知情同意。

2. 诊断及疗效评估：收集NDMM患者基线外周血及骨髓标本，骨髓标本经CD138单克隆抗体磁珠（德国美天旎生物技术公司产品）分选后，通过荧光原位杂交（FISH）技术进行检测，具体项目包括del（17p）（p53探针）、1q21+（S100A10探针）、del（13q）（RB1探针）及IgH重排。对于IgH重排阳性患者，进一步筛查t（4;14）（FGFR3探针）、t（11;14）（CCND1探针）、t（14;16）（MAF探针）和t（14;20）（MAFB探针）易位（探针购自广州安必平医药科技股份有限公司）。以健康供体的浆细胞作为对照，CA阳性阈值定义为：IgH重排≥10％，1q21+≥4.9％，del（13q）≥4.8％，del（17p）≥6.9％。研究将同时携带1q21+和t（4;14）的96例患者定义为DHMM，69例患者有克隆大小信息，66例（96％）1q21+克隆>20％，69例（100％）患者t（4;14）克隆>10％。92例（96％）患者基线行PET-CT检查。基线诊断时存在≥3个溶骨性病变的55例患者中，54例（98％）接受PET-CT检查，1例（2％）接受CT检查。22例sEMD患者的影像学检查方式包括PET-CT（6例）、CT（10例）、MRI（2例）和超声（1例）。MM的诊断及疗效评估均参照国际骨髓瘤工作组标准[13]。骨髓MRD监测频率：对于适合移植的患者，MRD的评估分别在诱导治疗4个周期后、auto-HSCT前、auto-HSCT后100 d内及随访期间。对于不适合移植的患者，MRD的评估在8次诱导治疗结束后。如果患者达到MRD阴性，前两年每6个月检测1次骨髓MRD状态，之后每年检测1次。通过二代流式细胞术检测骨髓MRD，灵敏度为10^−5^。

3. 治疗方案：治疗流程包括诱导治疗、auto-HSCT（适用于符合条件者）、巩固治疗（按需）及维持治疗。如前期研究所述[10]，诱导治疗采用PAD方案（4～8个周期），具体为：硼替佐米（第1、4、8、11天，1.3 mg/m^2^）、脂质体阿霉素（第1个周期连续3 d，总量30 mg/m^2^）、地塞米松（第1～4天，20～40 mg）。自2022年起，诱导治疗2个周期后未达部分缓解（PR）或携带高危CA［包括del（17p）、t（4;14）、t（14;16）］的患者，于第3～4个周期加用来那度胺（第1～21天，10 mg）和（或）达雷妥尤单抗（第1、8、15天，16 mg/kg）强化诱导治疗。诱导治疗后，符合auto-HSCT条件者于4个周期诱导治疗结束后采集造血干细胞，随后行auto-HSCT。auto-HSCT后100 d内评估疗效，未达完全缓解（CR）者接受4个周期额外巩固治疗。不符合auto-HSCT条件者需完成8个周期诱导治疗。维持治疗方案为来那度胺（第1～21天，10 mg）和（或）硼替佐米（每2周1.3 mg/m^2^），每28天为1个周期，最长持续2年。

4. 随访：本研究通过查阅门诊或住院病历及电话进行随访，随访截止至2025年3月，中位随访时间为36（6～126）个月。PFS期定义为患者自启动一线治疗至疾病进展或死亡的时间间隔，OS期定义为自启动一线治疗至死亡或末次随访的时间间隔。

5. 统计学处理：统计分析均基于R软件（版本4.4.2）完成，生存分析采用survival包（v3.8-3）进行，生存曲线可视化通过survminer包（v0.5.0）实现。定性资料用例数（百分比）表示，定量资料用*M*（范围）表示。变量间相关性分析采用Spearman秩相关系数。图形可视化（含散点图、箱线图等）均采用ggplot2包（v3.5.1）完成。连续变量的组间比较采用非配对双侧Wilcoxon秩和检验，分类变量的比较则根据数据特征选择Chi-squared检验或Fisher精确检验。多因素回归分析采用Cox比例风险模型进行，单因素分析中有统计学意义的变量纳入多因素分析，并结合临床相关性进行筛选，最终在模型中报告各变量的风险比（*HR*）、95％置信区间（*CI*）及*P*值。所有分析采用双侧检验，*P*<0.05为差异有统计学意义。

## 结果

1. 基线特征：患者中位年龄62（36～79）岁，男51例（53％）。疾病分期显示，35例（36％）为国际分期系统（ISS）/修订的国际分期系统（R-ISS）Ⅲ期，50例（52％）为R2-ISS Ⅳ期，提示高肿瘤负荷。细胞遗传学方面，11例（11％）合并del（17p），81例（90％）合并del（13q），无t（11;14）异常。临床症状表现为：17例（18％）伴高钙血症，30例（31％）存在肾功能损害（肾小球滤过率<60 ml/min），56例（58％）有贫血，14例（15％）伴血小板减少，55例（57％）存在广泛溶骨性病变（≥3个病灶），32例（33％）血清乳酸脱氢酶（LDH）水平升高。初诊时，14例（15％）患者伴EM-B，5例（5％）伴EM-E。39例（41％）患者接受PI、IMiD联合CD38单克隆抗体（CD38Ab）的三药诱导方案，35例（36％）接受auto-HSCT，40例（56％）接受PI联合IMiD两药维持治疗。最终，25例（26％）患者达到MRD阴性的CR。随访期间，22例（23％）患者发生sEMD，其中骨旁髓外复发（sEM-B）8例（8％），软组织髓外复发（sEM-E）14例（15％）。sEM-B最常累及椎骨旁区域［5例（63％）］，其次为胸骨［2例（25％）］和骨盆［1例（12％）］；sEM-E主要发生于软组织、皮肤、肌肉［5例（36％）］，胸膜及肺部［4例（29％）］，中枢神经系统［2例（14％）］和淋巴结［1例（7％）］，另有2例（14％）进展为浆细胞白血病。

2. 患者预后与克隆负荷的相关性：中位随访36个月，96例患者的中位PFS期为26（95％*CI*：22～50）个月，3年OS率为67％（95％*CI*：56％～79％），预估中位OS期为4.3（95％*CI*：2.1～6.4）年（图1）。克隆负荷分析显示，1q21+与t（4;14）的克隆大小呈显著正相关（*r*＝0.46，*P*<0.001，图2）。两种CA的克隆大小比较表明，t（4;14）克隆负荷高于1q21+（中位值：95.2％对87.3％，*P*<0.001，图3）。

**图1:**
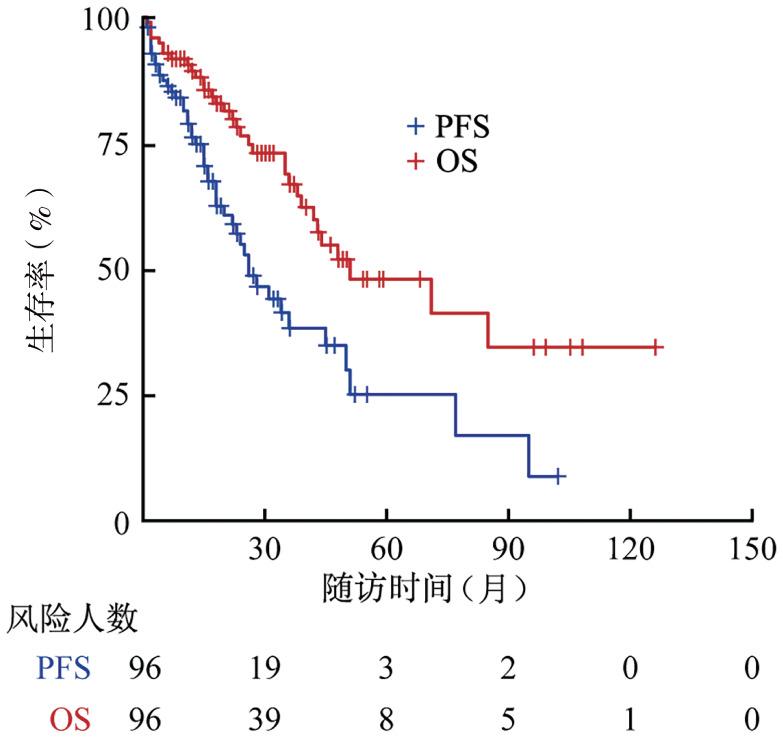
96例伴1q21+及t（4;14）双打击多发性骨髓瘤患者的无进展生存（PFS）和总生存（OS）曲线

**图2:**
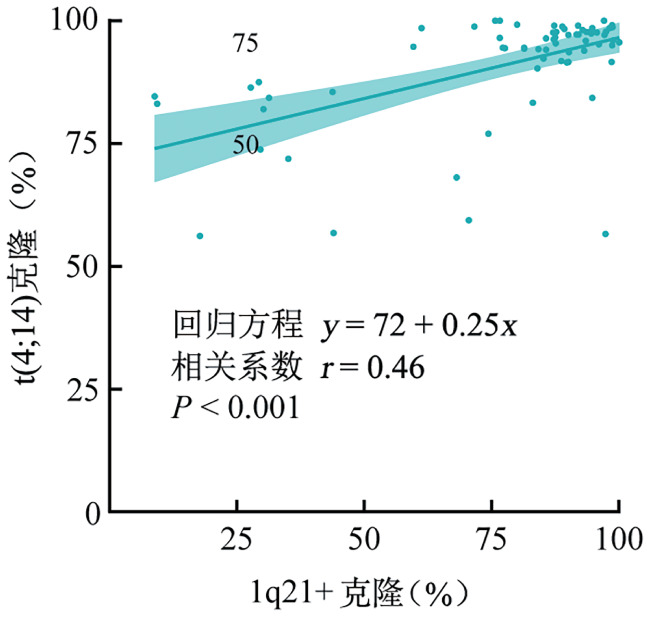
伴1q21+及t（4;14）的双打击多发性骨髓瘤患者1q21+与t（4;14）克隆的相关性

**图3:**
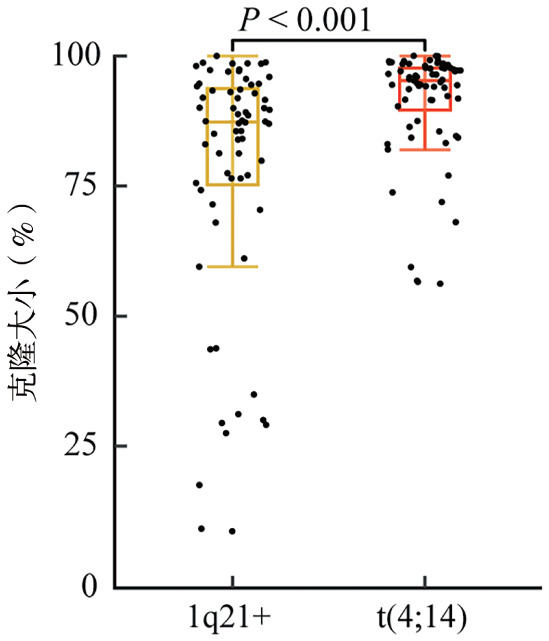
伴1q21+及t（4;14）的双打击多发性骨髓瘤患者1q21+与t（4;14）克隆大小

3. PFS和OS的单因素及多因素分析：PFS的单因素分析显示，年龄≥65岁（*HR*＝2.30，95％*CI*：1.25～4.22，*P*＝0.007）、del（17p）（*HR*＝2.79，95％*CI*：1.28～6.07，*P*＝0.010）、初诊伴EM-E（*HR*＝11.29，95％*CI*：3.98～32.03，*P*<0.001）显著缩短PFS期；auto-HSCT（*HR*＝0.35，95％*CI*：0.18～0.70，*P*＝0.003）、三联诱导治疗（PI+IMiD+CD38Ab）（*HR*＝0.34，95％*CI*：0.14～0.84，*P*＝0.019）、MRD阴性（*HR*＝0.12，95％*CI*：0.03～0.49，*P*＝0.003）显著延长PFS期；而性别、ISS/R-ISS分期Ⅲ期、R2-ISS分期Ⅳ期、1q21扩增、del（13q）、高钙血症、肾功能不全、贫血、血小板减少、美国东部肿瘤协作组（ECOG）评分>2分、初诊伴EM-B、两联诱导治疗、LDH升高、PI+IMiD联合维持治疗（与PI或IMiD或CD38Ab单药维持治疗比较）及2022年以后收治（与2014-2021年收治比较）等因素均与PFS无显著相关性（*P*值均>0.05）。

OS的单因素分析显示，年龄≥65岁（*HR*＝2.40，95％*CI*：1.18～4.86，*P*＝0.016）、ISS/R-ISS分期Ⅲ期（*HR*＝2.42，95％*CI*：1.22～4.80，*P*＝0.012）、del（17p）（*HR*＝2.47，95％*CI*：1.06～5.75，*P*＝0.035）、肾功能不全（*HR*＝2.59，95％*CI*：1.28～5.25，*P*＝0.008）、高钙血症（*HR*＝2.45，95％*CI*：1.16～5.19，*P*＝0.019）、初诊伴EM-E（*HR*＝11.28，95％*CI*：3.40～37.44，*P*<0.001）及EM-B（*HR*＝4.19，95％*CI*：1.57～11.13，*P*＝0.004）显著增加死亡风险；auto-HSCT（*HR*＝0.32，95％*CI*：0.13～0.79，*P*＝0.013）、MRD阴性（*HR*＝0.11，95％*CI*：0.02～0.84，*P*＝0.033）显著改善OS，三联诱导治疗（PI+IMiD+CD38Ab）有改善OS的趋势（*HR*＝0.29，95％*CI*：0.08～1.03，*P*＝0.055）；而性别、R2-ISS分期Ⅳ期、1q21扩增、del（13q）、贫血、血小板减少、ECOG评分>2分、两联诱导治疗、LDH升高、PI+IMiD联合维持治疗（与PI或IMiD或CD38Ab单药维持治疗比较）及2022年以后收治（与2014-2021年收治比较）等因素与OS均无显著相关性（*P*值均>0.05）。

将单因素分析中有统计学意义的因素纳入多因素分析，结果显示，初诊伴EM-E（*HR*＝7.25，95％ *CI*：2.46～21.39，*P*<0.001）和del（17p）（*HR*＝2.59，95％*CI*：1.12～5.98，*P*＝0.025）是PFS期缩短的独立预后因素，而MRD阴性（*HR*＝0.18，95％*CI*：0.04～0.90，*P*＝0.037）是PFS期延长的独立预后因素（表1）。初诊伴EM-E（*HR*＝5.70，95％*CI*：1.43～22.73，*P*＝0.014）和EM-B（*HR*＝3.69，95％*CI*：1.23～11.10，*P*＝0.020）均是OS的独立不良预后因素（表1）。

**表1:** 影响96例伴1q21+及t（4;14）双打击多发性骨髓瘤患者无进展生存和总生存的多因素分析

因素	无进展生存		总生存	
	*HR*（95％*CI*）	*P*值	*HR*（95％*CI*）	*P*值
伴del（17p）	2.59（1.12～5.98）	0.025	2.11（0.81～5.50）	0.129
伴EM-B	2.46（0.96～6.30）	0.061	3.69（1.23～11.10）	0.020
伴EM-E	7.25（2.46～21.39）	<0.001	5.70（1.43～22.73）	0.014
MRD阴性	0.18（0.04～0.90）	0.037	0.21（0.03～1.68）	0.141

**注** EM-B：骨旁髓外病变；EM-E：软组织髓外病变；MRD：微小残留病

4. MRD阴性的影响因素：本研究证实，MRD阴性状态是改善PFS的独立预后因素。接受auto-HSCT患者的MRD阴性率显著高于未接受auto-HSCT患者（51.4％对13.0％，*P*<0.001）。对诱导治疗方案的分析显示，PI+IMiD+CD38Ab三药联合方案组的MRD阴性率高于单药PI方案组及PI+IMiD双药方案组（51.3％对8.8％对8.7％，*P*<0.001）。多因素回归分析显示，三药诱导治疗（*OR*＝12.50，95％*CI*：3.19～67.50，*P*＝0.001）和auto-HSCT（*OR*＝9.64，95％*CI*：3.03～36.13，*P*<0.001）是患者MRD阴性的独立预后因素。

## 讨论

本团队既往单中心研究报道，NDMM患者的中位OS期为7.7年[11]，该研究比较了无异常（193例）、单独1q+（189例）、单独t（4;14）（18例）、1q+合并t（4;14）（53例）四组患者的预后，中位PFS期分别为41个月、24个月、56个月、18个月（*P*<0.05）；3年OS率分别为89％、88％、82％、73％（*P*<0.05），提示1q21+合并t（4;14）患者预后最差。本研究从更大样本量的NDMM患者中发现伴1q21+合并t（4;14）的DHMM患者3年OS率仅67％（预估中位OS期4.3年），表明即便在新药和auto-HSCT时代，该亚组患者预后仍不良。基线特征方面，DHMM患者EMD的发生率与既往本中心报道的NDMM患者相似[14]，其中EM-B占14％，EM-E占6％。细胞遗传学分析显示，1q21+与t（4;14）克隆大小呈正相关，提示两种异常事件并非随机共存。t（4;14）克隆中位占比超过95％，提示其可能作为疾病发生的始发驱动事件，而1q21+克隆中位数为87％，或为继发演变结果。值得注意的是，DHMM患者的1q21+克隆显著高于MM患者，与既往研究得出的“较大1q21+克隆与合并其他高危CA相关”结论一致[15]。

本团队前期研究已证实，携带≥2个高危CA的MM患者易发生继发性EMD[11]。本研究中DHMM亚组3年中位随访显示，sEMD率达23％，显著高于618例NDMM患者（10％）[11]，与美国梅奥诊所报道的“携带1q21+或t（4;14）的NDMM患者更易发生sEMD”结论一致[16]。多因素分析进一步表明，初诊伴EM-E和del（17p）是PFS的独立危险因素，而初诊伴EMD（包括EM-E和EM-B）则显著缩短OS期。上述结果提示，DHMM患者如果基线存在额外的del（17p）形成“三打击”状态或合并EMD，需采用更强化的诱导、巩固及维持治疗以改善患者预后。

本研究显示，MRD阴性可独立显著改善伴1q21+及t（4;14）的DHMM患者的PFS。多因素分析显示，auto-HSCT与三联诱导治疗（PI+IMiD+CD38Ab）均为MRD阴性的独立预后因素，支持两者同时应用的治疗策略。这一结果与GRIFFIN和ISKIA研究一致[17]–[18]，表明在以PI和IMiD为基础的诱导方案中加入CD38Ab可进一步提高适合移植DHMM患者的MRD阴性率。然而，MRD阴性不能独立降低死亡风险，获得MRD阴性的DHMM患者仍可能出现疾病进展或死亡，与MASTER研究结果一致[19]。本团队的真实世界研究也显示，DHMM患者移植后MRD阴性状态的1年丢失率高达41％，显著高于无高危CA（7％）或有1个高危CA（19％）的NDMM患者[10]。本研究对单药维持治疗和双药维持治疗的PFS进行比较（*HR*＝1.96，95％*CI*：0.91～4.21，*P*＝0.086），提示双药维持治疗的PFS有获益趋势。此外，本研究25例获得MRD阴性的患者中，20例接受维持治疗，且均采用PI联合IMiD双药维持治疗方案。结果显示，1年持续MRD阴性率为79.5％，1年MRD阴性丢失率为20.5％，低于本中心历史对照患者（41％），可能是由于15例获得MRD阴性的历史对照DHMM患者中10例采用单药维持治疗（PI或IMiD）。本团队真实世界数据均提示，DHMM患者达到MRD阴性后仍需强化维持治疗。Ⅱ期FORTE研究发现，高危MM患者维持治疗中采用卡非佐米+来那度胺（KR方案）较单用来那度胺更具生存获益趋势[20]–[21]。值得注意的是，GRIFFIN研究DaraVRD方案组中仅20％的DHMM患者实现持续MRD阴性（阈值为10^−5^，≥12个月）[17]，FORTE研究中该比例为24％[21]，提示在接受新药治疗的临床研究中，DHMM预后不良可能与无法维持持续MRD阴性有关。因此，针对DHMM患者，需采用可实现持续MRD清除的治疗方案，并进行长期序贯的MRD深度监测。新兴的靶向B细胞成熟抗原（BCMA）的嵌合抗原受体T（CAR-T）细胞疗法如西达基奥仑赛（cilta-cel）和伊基奥仑赛（eque-cel）已在复发难治性MM（RRMM）中展现潜力，分别使55％和74％的RRMM患者实现持续MRD阴性（阈值为10^−5^，≥12个月）[22]–[23]。本团队前期研究对接受cilta-cel治疗的RRMM患者进行长期随访，MRD阴性率达67.6％，5年OS率为49.1％[24]。提示CAR-T细胞疗法的持续MRD清除能力可能改善患者生存，其在DHMM前线治疗中具有潜在应用价值。

本研究存在部分局限性，如为回顾性研究、诱导治疗方案存在异质性等，但这一特点也为观察不同方案组合对MRD状态及预后的影响提供了独特视角。通过对单中心96例携带1q21+和t（4;14）的DHMM患者进行分析，本研究系统揭示了该亚群患者的临床特征、预后及影响预后的因素，显示三联诱导治疗（PI+IMiD+CD38Ab）及auto-HSCT在实现MRD阴性中的有效性，强调维持MRD阴性的重要性。值得注意的是，国际骨髓瘤学会/国际骨髓瘤工作组发布的高危MM定义中，已将1q+合并t（4;14）列为高危特征，而单独的1q+或t（4;14）不再归为高危[25]。反映了随着治疗模式的创新与发展，传统DHMM的超高危特征已逐步被弱化为高危。尽管如此，该领域仍存在未被满足的临床需求，有待进一步研究探索。
